# Salutatory

**Published:** 1867-01

**Authors:** Jno. M. Johnson


### EDITORIAL AND MISCELLANEOUS.

### SALUTATORY.

I take this method of announcing to the friends and patrons of the Atlanta Medical and Surgical Journal, that I have become associated with Professors J. G. & W. F. Westmoreland, in the editorial management of it. It is not without an unfeigned distrust of my qualifications that I have done so; nor can I hope to equal, much less to excel my co-laborers in the field, thus newly opening before me. But I am impelled by an unaffected love of the profession of medicine, to which I have devoted a life not now short, with earnest zeal, and all the capacity nature has endowed me with.

My achievements in the profession, and my opinions, may be pronounced by those better qualified to judge them, a failure. Be this as it may, I am moved by a sense of duty, to give to the brotherhood of medicine, an epitome at least, of my long experience and observation, with the hope that my failures, if not my successes, may be of some use to the more youthful devotees to medicine, in inducing them to shun the quicksands of error, and false theories, the every day stumbling block of the idle and lazy, and of exalting their ambition to a just appreciation of the lofty dignity of the profession, as illustrated by Rush, and Physic, and Chapman, and Drake, and Eberle, and thousands of others living and dead, whose names will shine forever upon the canvass of time. No matter how well he may understand the elementary principles of medicine, it requires great energy and industry, practical wisdom and judgment, and inflexible honesty, to enable the Physician to stand acquitted at the forum of conscience, where every word and work should be tried.

By the consent of all enlightened people, the Physician holds an exalted place in society. He is not only a prefered guest of the household, but is received into the inner temple of their hearts, and intrusted with the most delicate duties and privileges. If his integrity remains spotless, and all his doings shall have been dictated by an enlightened judgment, he can stand at the close of such a life, as if upon an eminence, and looking back upon the pleasing picture, say with the inspired man, "I have fought a good fight."

This Journal will for the future, as in the past, be open to all Medical Philosophers, who may desire to communicate their views to their brethren, and contributions are respectfully solicited.

The late unfortunate civil war, whilst it has begerted and panoplied our land with curses, has not been without compensating results, so far as Medicine and Surgery are concerned. The doubtful problem of Resections, Wounded Joints, Hospital Gangrene, Piemia, &c., &c., but little discussed before the war, and still less understood, are now widely known. Men of small pretensions in this important branch of science then, are to-day knocking at the door of fame, and well do they deserve the honors they have won.

In Medicine, as in Surgery, the progress is magical. Humoralists and solidists have alike given up their hobbies, and embraced the great idea, that the Cerebro-Spinal system is the centre of morbid energy—the source of organic election, and the portal that leads on to pathological truth. The rubbish of centuries is being cleared away—the magical influence of standard authorities and names, is rapidly becoming a part of the great past. Whilst the sheen of glittering generalities—the bane of schools and the enemy of true progress—becomes "their air," before the experience of twenty thousand Medical Philosophers, each armed with his record of facts. A new era in Medicine and Surgery dawned with the war, to eclipse in benefits to mankind, let us hope the fame of its mighty chiefs. Let us snatch from the ruin one trophy at least, and for this purpose, we appeal to the brotherhood of medicine, North and South, to be true to themselves and to humanity, in laying the foundation of progress broad and deep, to the honor of the present and the glory of the future of our noble profession.

JNO. M. JOHNSON, M. D.

